# Coarse Breads

**Published:** 1873-06

**Authors:** 


### COARSE BREADS

Are made of the product of the whole grains ground into the form of meal; when the coarser particles are removed it is called flour. Corn meal makes corn bread; wheat meal makes graham, or brown bread, because it contains the "bran" or outer hull of the wheat grain, but this bran has attached to it three-fourths of all the real nutriment of the grain of wheat, the part which makes bone and solid teeth and hard flesh. The coarser preparations of all the grains, whether ground up in the form of meal, or broken into small pieces, making hominy if of corn, or crushed wheat if of that form of grain, are more healthful than the much finer forms of flour, not only because they contain more nourishment, but because they have a tendency to promote the regular operations of nature, as "roughness," or fodder, or hay or coarse grass, in the case of horses. Fruits, berries, grapes, and coarse breads as principal articles of food, if persevered in, would "regulate" multitudes who now rely most injuriously on pills, salts, mineral waters and enemas, the tendencies of all of which are ruinous to any constitution.

### OATMEAL.

One of the best methods of preparing oatmeal is to boil it thus: Put four table-spoonfuls of the meal in a saucepan with a little salt, pour on near a pint of boiling water, stir it well and place it on the stove, continue the stirring from time to time until it begins to thicken, then let it slowly boil for twenty minutes, to be eaten with butter, sugar, syrup, or milk. Wheat meal or graham flour makes

### BROWN BREAD

thus: Pour a quart of boiling water on a teacupful of wheat meal, stir it well and when a little boiled stir in a heaping teaspoonful or more of salt, and as much brown sugar, add a heaping teaspoonful of fresh pork lard, then two tablespoonfuls of yeast and as much flour as can be stirred in with a spoon or paddle; let it rise in a warm place all night, pour the dough into a greased cast-iron pan, and bake within an hour.

### BREAKFAST CAKES.

Let a pint of oatmeal stand in a quart of water, overnight, then add a teaspoonful of salt, a tablespoonful of sugar and as much baking powder, then stir in a pint of fine or graham flour; make it thinner for griddle cakes, thicker for muffins.

### OAT BREAD.

Stir a quart of oatmeal in two quarts of water, let stand six or eight hours, then stir in a quart of flour, two or three tablespoonfuls of sugar, and two tablespoons of baking powder.

### MUSH AND PORRIDGE.

The much-loved mush of the Southerner, and the dearly beloved porridge of the Scotchman, are one and the same thing, and prepared in the same manner by thorough boiling, only the mush is made of corn meal, and the porridge is made of oatmeal.

### OATMEAL PIE

may be eaten to advantage by the most discouraged dyspeptic, if prepared properly. Stir four tablespoonfuls of oatmeal into a pint of water, let stand until the meal is thoroughly swelled, add two or three sliced apples, with sugar and spice to suit, and bake as an ordinary pie.

### BARLEY BISCUITS

are made of equal parts of barley meal and fine wheat flour with water and "rising" and salt to suit.

### WHEAT MEAL BREAD.

Stir a pint of wheat meal, called graham flour, into a pint of cold water, and mash up the little lumps until none are left, then stir in three or four tablespoons of cold water; this makes a batter, pour this into cast iron pans, hot and greased, put in a hot oven, so as to cook within forty minutes; take them out, wrap them up well in a cloth for half an hour; when they are cooled, soft and light, ready for eating alone or with butter. The above mixture is said by an enthusiastic hydropathist to make "eleven small loaves of bread, weighing in all one and a half pounds. I eat four of them at a meal on an average; upon this I have lived almost exclusively for more than three years with a moderate amount daily of wheat meal pudding, rice, corn meal, and fruits and vegetables, nothing else. I endure heat and cold better than ever before, have increased in vigor of mind and body; am not subject to colds, headaches, indigestion, neuralgia, or fevers; often eat but once a day, and have no longings, craving, gnawings, 'goreness,' achings or heaviness of stomach." There can be no doubt that many dyspeptics could cure themselves by a similar diet, thrice a day, with lean meat for dinner and a seasonable daily exposure in out-door activities of a pleasurable or encouragingly remunerative character. No medicine known can possibly cure dyspeptics; it must be done by the regulation of the food, so as to adapt it to the requirements of the system and the capabilities of the stomach.

